# Indoor Temperatures in Low Cost Housing in Johannesburg, South Africa

**DOI:** 10.3390/ijerph14111410

**Published:** 2017-11-18

**Authors:** Nisha Naicker, June Teare, Yusentha Balakrishna, Caradee Yael Wright, Angela Mathee

**Affiliations:** 1 Environment and Health Research Unit, South African Medical Research Council, Johannesburg 2028, South Africa; june.teare@mrc.ac.za (J.T.); cwright@mrc.ac.za (C.Y.W.); angela.mathee@mrc.ac.za (A.M.); 2 Environmental Health Department, Faculty of Health Sciences, University of Johannesburg, Johannesburg 2028, South Africa; 3 The Epidemiology and Surveillance Section, National Institute for Occupational Health, National Health Laboratory Services, Braamfontein 2001, South Africa; 4 Department of Environmental Health, School of Behavioural Sciences, Faculty of Health Sciences, Nelson Mandela University, Port Elizabeth 6019, South Africa; 5 Biostatistics Unit, South African Medical Research Council, Durban 4091, South Africa; yusentha.balakrishna@mrc.ac.za; 6 Department of Geography, Geoinformatics and Meteorology, University of Pretoria, Pretoria 0028, South Africa; 7 Wits School of Public Health, Faculty of Health Sciences, University of Witwatersrand, Johannesburg 2193, South Africa

**Keywords:** climate change, environmental health, urban, heat, cold, low cost housing, indoor temperature, ambient temperature

## Abstract

Ambient and indoor temperature affects thermal comfort and human health. In a changing climate with a predicted change in temperature extremes, understanding indoor temperatures, both hot and cold, of different housing types is important. This study aimed to assess the hourly, daily and monthly variation in indoor temperatures in different housing types, namely formal houses, informal houses, flats, government-built low-cost houses and old, apartheid era low-cost housing, in five impoverished urban communities in Johannesburg, South Africa. During the cross-sectional survey of the Health, Environment and Development study data loggers were installed in 100 homes (20 per suburb) from February to May 2014. Indoor temperature and relative humidity were recorded on an hourly basis. Ambient outdoor temperatures were obtained from the nearest weather station. Indoor and outdoor temperature and relative humidity levels were compared; and an inter-comparison between the different housing types were also made. Apparent temperature was calculated to assess indoor thermal comfort. Data from 59 retrieved loggers showed a significant difference in monthly mean indoor temperature between the five different housing types (*p* < 0.0001). Low cost government-built houses and informal settlement houses had the greatest variation in temperature and experienced temperatures between 4 and 5 °C warmer than outdoor temperatures. Housing types occupied by poor communities experienced indoor temperature fluctuations often greater than that observed for ambient temperatures. Families living in government-built low-cost and informally-constructed homes are the most at risk for indoor temperature extremes. These types of housing should be prioritised for interventions aimed at assisting families to cope with extreme temperatures, gaining optimal thermal comfort and preventing temperature-related health effects.

## 1. Introduction

While an inextricable link has been established between housing quality and health [1,2], relatively little is known about thermal comfort and health specifically, especially in low-income dwellings [3]. House characteristics, such as insulation, age of the dwelling, ceiling and the pitch of the roof, play defining roles in indoor thermal comfort [4]. Passive cooling strategies to improve thermal performance include window positioning to reduce heat gain and aid natural ventilation, external shading around the house, for example natural vegetation, blinds and awnings and the use of reflective roof colours [5]. Thermal comfort may take on increasing health significance in the light of rising temperatures and an increasing frequency of heat or cold extremes associated with climate change. Extreme temperatures have known adverse acute and chronic impacts on human health and have been directly associated with increased mortality [6,7].

Informal settlement dwellings, at risk of thermal comfort challenges, are semi-permanent structures built from sheets of corrugated iron, bricks, wood, and plastic. In South Africa, about 11% of the urban population live in informal settlements [8]; about 30% of these households have members who have submitted their names to receive low cost housing built by the government. Thus, this group of residents are extremely vulnerable to variations in ambient temperature. In addition, low cost housing has been characterized by dwellings with single walls that are usually too hot in summer and too cold in winter when exposed to prolonged hot or cold weather conditions. Cavity walls, i.e., walls formed from two thicknesses of brickwork with a space between them and particularly insulated cavity walls provide protection from external temperatures and can double as a moisture barrier [9]. Thermal inefficiency in low cost housing due to poor ventilation has also been noted [10] possibly due to the lack of air bricks (a perforated brick which is built into the wall and is used for ventilation), as reported by some residents, and windows kept open for ventilation, resulted in dust accumulating in the houses [11,12]. This presents a health hazard especially for those living in government-built housing close to mine dumps [13]. Since 1994 (new government, post-apartheid), consumer satisfaction surveys regarding the quality of post-1994 low cost housing have revealed a multitude of complaints resulting in dwelling repairs totaling R2.1 billion for the period 2011–2014 [14]. In April 2014, a new Norms and Standards for housing was approved [15]. Improvements should include larger dwellings (at least 40 m^2^), insulated ceilings, and plastered interior walls [15], which are lacking in low cost dwellings built prior to 2015. 

Global climatic projections show that surface (ambient) temperatures will increase more rapidly over Africa than elsewhere [16]. The City of Johannesburg, South Africa, is expected to experience an average rate of increase of 1.1 hot days per year where hot days are defined as having an average apparent (‘real-feel’ calculation combining temperature and relative humidity) temperature of ≥27 °C; for the time period 2011 to 2040 an average of 69.5 hot days per year have been predicted [17]. Increases in the intensity and frequency of heat waves have been linked to increases in heat-related morbidity from cardiovascular related diseases and all-cause mortality [18,19,20]. Vulnerable groups include the elderly, children and babies [21,22,23], as well as low-income households, people residing in urban areas, and the homeless [3,23,24].

Coping with, and adapting to, the effects of climate change is influenced by factors such as wealth, education, infrastructure, access to information, resources and technology, and management skills [25]. Thus, low-income communities are particularly disadvantaged in relation to coping with, recovering from, and adapting to climate change and its ill-health effects [3]. A study conducted in households in a Western Cape township consisting of informal and formal houses that assessed indoor wintertime temperatures, found that in indigent homes indoor temperatures can be up to 5 °C lower than outdoor temperatures [26], resulting in a need for affected families to spend money on coal in order to improve indoor thermal comfort. No study was identified that considered indoor temperatures in indigent, urban communities in South Africa. Hence, this study aimed to assess the daily and monthly variation in indoor temperatures in different housing types, namely formal and informal houses, flats (apartments), post 1994 government-built low cost (Reconstruction and Development Programme [RDP] housing) and pre-1994, apartheid era low-cost housing, in impoverished urban communities in Johannesburg, South Africa.

## 2. Materials and Methods 

### 2.1. Study Population and Sample 

**Figure 1:**
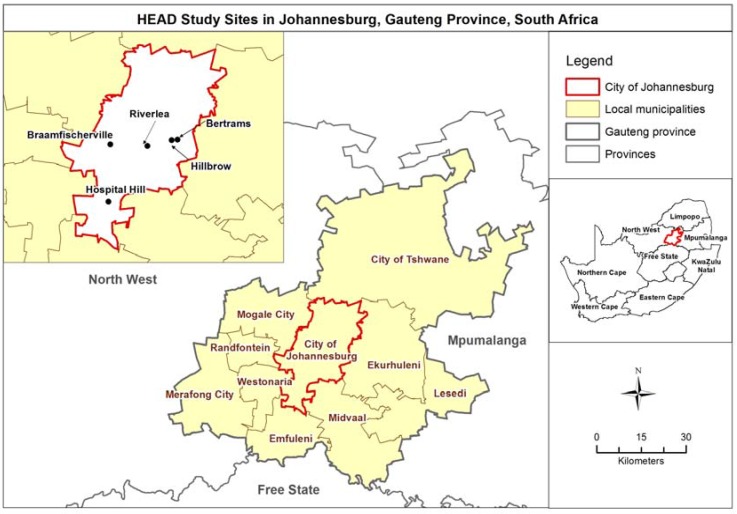
Location of the five study sites in the City of Johannesburg, South Africa.

This study was conducted in a randomly selected sub-sample of houses included in the Health, Environment and Development (HEAD) panel study [27]. The HEAD study is an annual, cross-sectional survey conducted in five settlements in the City of Johannesburg, South Africa (Figure 1). The aim of the HEAD study was to monitor changes in living conditions in households and health status of household residents in five sentinel sites over a 10-year period from 2006 to 2016. Table 1 describes the types of housing found in impoverished areas in Johannesburg, South Africa. These housing types are represented in the five study sites included in this study. 

Except for Hospital Hill, where only a few dwellings were connected to electricity, all other sites had access to electricity, indoor water source and sanitation. Appliances included refrigerators and electric stoves. Air conditioning was not available in any of the dwellings [27]. Unfortunately, information on other cooling devices e.g., fans was not obtained in this study. Heating was mainly via electric heaters in the four study sites and burning of fossil fuels in Hospital Hill. Insulation was not present in any of the dwellings. The majority of dwellings had one window per room.

In the main study two hundred dwellings were selected in each site. In Braamfischerville, Riverlea, and Bertrams, dwellings were randomly selected using a table of random numbers and town planning maps of the study areas. In Hillbrow, apartment buildings were first randomly selected, followed by floors in the selected buildings and then apartments on the selected floors. In Hospital Hill, the informal settlement, convenience sampling was undertaken due to the lack of maps and formal addresses. In 2014, a sub-sample of 20 dwellings per site was randomly selected from the original HEAD study sample to assess indoor temperature.

### 2.2. Data Collection

Data on housing type and materials were collected through administration of a pre-structured questionnaire by researchers from the South African Medical Research Council and University of Johannesburg. Only one adult member, 18 years or older, from each household was interviewed. Ethical approval for this study was obtained from the University of the Witwatersrand Human Research Ethics Committee (Research Ethics Approval number: M10471). Questionnaires were administered after obtaining written, informed consent from respondents. 

Battery operated LogTag, Haxo-8 indoor temperature monitors were placed in the living room in the houses to measure air temperature and relative humidity (RH). The loggers are capable of measuring air temperature from −40 °C to +80 °C and relative humidity from 0 to 100% RH. The devices were calibrated by the manufacturer and temperature calibration was not necessary. The RH sensor should be calibrated every 6 to 12 months depending on the environment it is placed in. The devices were placed in all the selected dwellings, at a location away from direct sunlight and heat, and on a dry surface from 18 February 2014 (end of austral summer) to 12 May 2014 (mid-autumn). They were programmed to record data at hourly intervals throughout the day. At the end of the study period, the loggers were collected and data were downloaded using a USB cable. Data were analysed using the LogTag Analyser software. Ambient, outdoor temperature and humidity readings for the same period were obtained for the Botanical Garden (−26.1560 S; 27.9990 E) monitoring station in the City of Johannesburg from the South African Weather Service (SAWS). This SAWS monitoring station is within a 10 km radius of Riverlea, Bertrams and Hillbrow and within 20 km of Braamfischerville and 38 km from Hospital Hill.

### 2.3. Apparent Temperature Calculations

In addition to measured temperature, we also calculated apparent temperature (*AT*) (e.g., ‘real-feel’ conditions) using indoor temperature and RH measurements made in the houses. Apparent temperature is an indicator of thermal sensation, can be used in indoor settings [28] and has been used before when considering the relationship between heat and thermal comfort [29,30]. *AT* was calculated using the temperature and RH measurements made by the data loggers and using Equation (1):

*AT* = *Ta* + 0.33 × *e* − 0.70 × *ws* − 4.00 (1)

where *AT* is apparent temperature, *Ta* is measured dry bulb temperature (°C) in the house, *e* is water vapour pressure (hPa) and *ws* is wind speed [31]. Given that this is an indoor setting, *ws* was set to 0. Water vapour pressure was calculated using the RH measurements made in the houses and applying Equation (2) as follows:

*e* = *RH*/100 × 6.105 × exp(17.27 × *Ta*/(237.7 + *Ta*)) (2)

where *RH* is relative humidity (%). The *AT* calculations were made for comparison and discussion purposes in relation to the measured indoor and ambient outdoor temperatures.

### 2.4. Data Analysis

Data were analysed using Stata version 14 (StataCorp., College Station, TX, USA). Monthly mean (with standard deviation and range) indoor and ambient temperatures and RH measurements were calculated by housing type according to the different study sites. Hourly mean indoor measured and apparent temperatures and RH were also calculated by housing type and graphically displayed. Differences in temperatures between housing types were tested using one-way ANOVA and a pairwise comparison between each site was performed. Pearson’s correlation (*r*) was used to quantify the correlation between indoor and ambient temperatures and the relationship was examined using linear regression. The relationship between indoor and ambient RH was examined using fractional polynomials. Categorical variables were described using frequencies and percentages. The impact of housing features, such as the floor, wall and ceiling material, on indoor measured and apparent temperature were investigated using a time adjusted two-level multilevel regression model with a random intercept and slope for each household and day. This was conducted to control for the lack of independence between measurements in homes. Results were considered statistically significant for *p*-values less than 0.05.

## 3. Results

### 3.1. Sample Description

One hundred data loggers measuring temperature and RH were installed in the dwellings across the five suburbs. Results were obtained for 59 dwellings: 10 formal houses in Bertrams, 15 RDP houses in Braamfischerville, 13 pre-1994-low cost homes in Riverlea, 13 flats in Hillbrow and eight informal dwellings in Hospital Hill. In 41 dwellings, the devices could not be recovered due to their having been lost, sold, tampered with or broken. 

In Hillbrow, Bertrams and Riverlea dwellings were approximately 50 years or older, while in Braamfischerville the average age of dwellings was 14 years. Informal housing in Hospital Hill consisted of temporary structures constructed from a variety of materials for flooring, ceilings and walls, which may be readily dismantled. In most instances, the dwellings were without a foundation, and constructed directly upon the soil, without being secured. As can be seen from Table 2, the majority of dwellings in the informal settlement of Hospital Hill, as well as in the RDP housing settlement of Braamfischerville, were not fitted with a ceiling.

### 3.2. Indoor Household Temperatures

**Figure 2:**
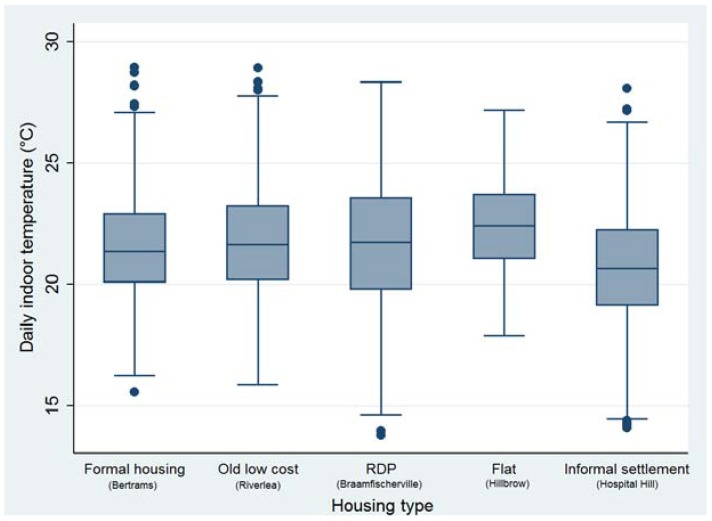
Box plot of daily indoor temperature by housing type. Top whisker: greatest value excluding outliers; Upper quartile: 25% of the data greater than this value; Median: 50% of data is greater than this value; Lower quartile: 25% of the data are less than this value; Bottom whisker: minimum value excluding outliers.

Table 3a provides a summary of the temperatures experienced within the homes of each area for each month of the study period. There was a significant difference in monthly mean indoor temperature between housing types (*p* < 0.0001). The differences between minimum and maximum temperatures are large especially in Riverlea, Braamfischerville and Hospital Hill. Figure 2 shows that Braamfischerville (RDP) houses and Hospital Hill (informal settlement) houses had the greatest variation in indoor temperature, suggesting that these structures are most sensitive to changes in ambient outdoor temperature. Pairwise comparisons between each site was conducted and in February the pairwise comparisons were significantly different from each other (*p* ≤ 0.001). In March all pairwise comparisons were significantly different from each other (*p* < 0.001) except for Riverlea/Braamfischerville (*p* = 0.112), Hillbrow/Braamfischerville (*p* > 0.999), and Riverlea/Hillbrow (*p* = 0.052). In April all pairwise comparisons were significantly different from each other (*p* < 0.001 and *p* = 0.026 for Bertrams/Riverlea) except for Bertrams/Braamfischerville (*p* > 0.999). In May all pairwise comparisons were significantly different from each other (*p* < 0.001) except for Riverlea/Braamfischerville (*p* > 0.999).

Table 3b provides a summary of the apparent temperatures experienced within the homes of each area for each month of the study period. Indoor temperatures were significantly different from apparent temperatures for all housing types for all months (*p* < 0.001), except for RDP houses in Braamfischerville in April (*p* = 0.196).

**Figure 3:**
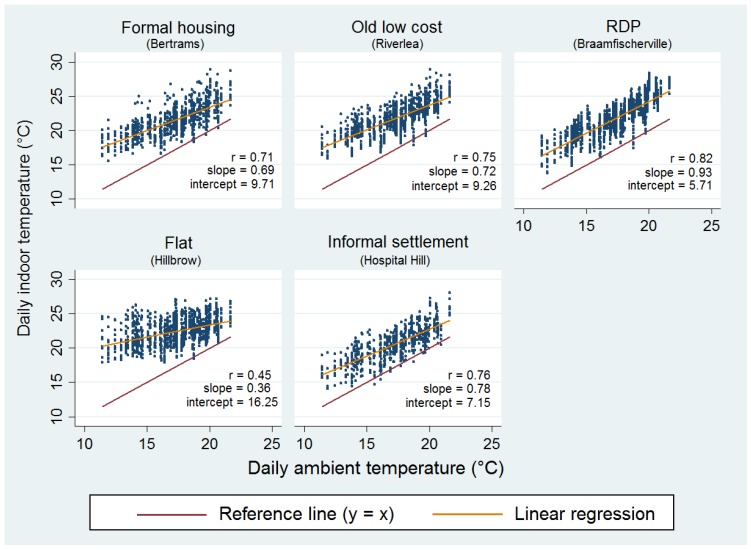
Regression results of daily mean indoor versus ambient outdoor temperatures per housing type found in the five study sites.

Indoor temperatures were significantly higher (> ~4 to 5 °C) than ambient temperatures for all areas (*p* < 0.001) over the study period (Figure 3). The red line represents the y = x reference line (slope = 1) i.e., when indoor temperature was equal to ambient temperature. The graph shows that Braamfischerville housing indoor temperatures were most strongly correlated (*r* = 0.82) to ambient temperature since the fitted linear line has a similar slope to that of the reference line followed by Hospital Hill at *r* = 0.76. The indoor temperatures of apartments in Hillbrow were least correlated (*r* = 0.45) with ambient temperature, suggesting that ambient temperature is a weak indicator of indoor temperatures in apartments. 

Participants were asked what the interior of the house felt like when it was hot outside using the ASHRAE (American Society for Heating, Refrigerating and Air-Conditioning Engineers) thermal comfort scale. In Braamfischerville (67%), Riverlea (54%) and Bertrams (70%) more than two-thirds of the participants felt uncomfortably hot or hot when it was hot outside. In Hillbrow and Hospital Hill only 39% and 38%, respectively, felt that the interior was uncomfortably hot.

**Figure 4:**
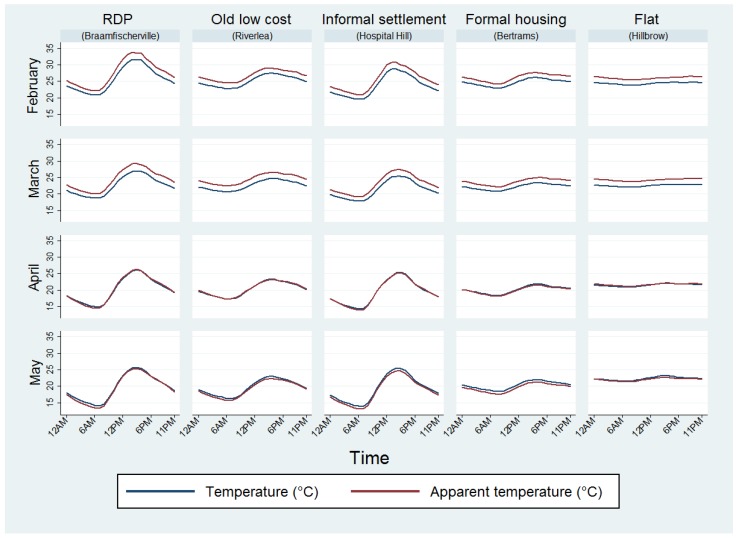
Mean measured and apparent temperatures experienced throughout the day.

When assessing hourly temperature over the four-month period, informal settlements and RDP dwellings experienced the greatest variation whilst formal housing in Bertrams and flats remained at relatively constant temperatures throughout the day (Figure 4). The lowest temperatures were experienced just after 06:00 and the highest temperatures were experienced during mid-afternoon. Apparent temperatures were higher than measured temperatures for all housing types during February and March but dropped slightly below measured temperatures in May.

### 3.3. Indoor House Relative Humidity Levels

Table 4 provides a summary of the RH levels experienced within each housing type for each month of the study period. There was a significant difference in humidity between housing types for all months (*p* < 0.0001).

**Figure 5:**
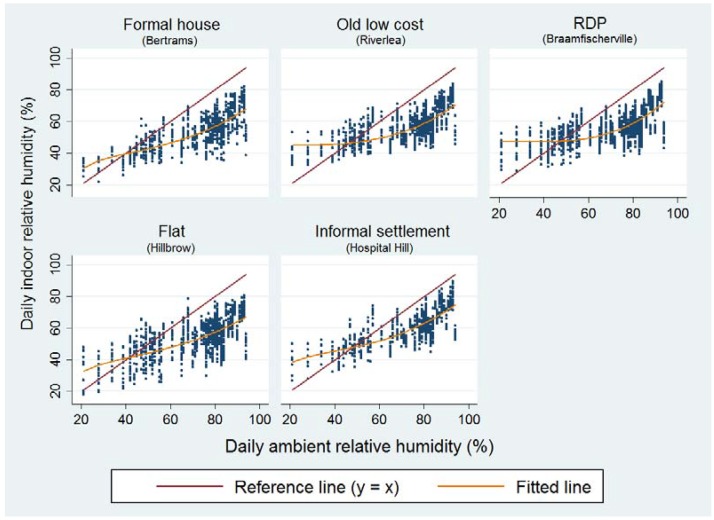
Daily mean relative humidity per housing type found in the five study sites. The reference line indicates a 1:1 match between indoor and ambient outdoor relative humidity (RH) values. The fitted line illustrates the deviation from the reference line.

Mean indoor RH was generally lower than ambient humidity after a certain threshold for all housing types but still relatively high (>49.0%). Indoor humidity dropped below ambient RH at approximately 40% ambient RH for formal housing and flats while the other housing types dropped after 40% ambient RH (Figure 5).

**Figure 6:**
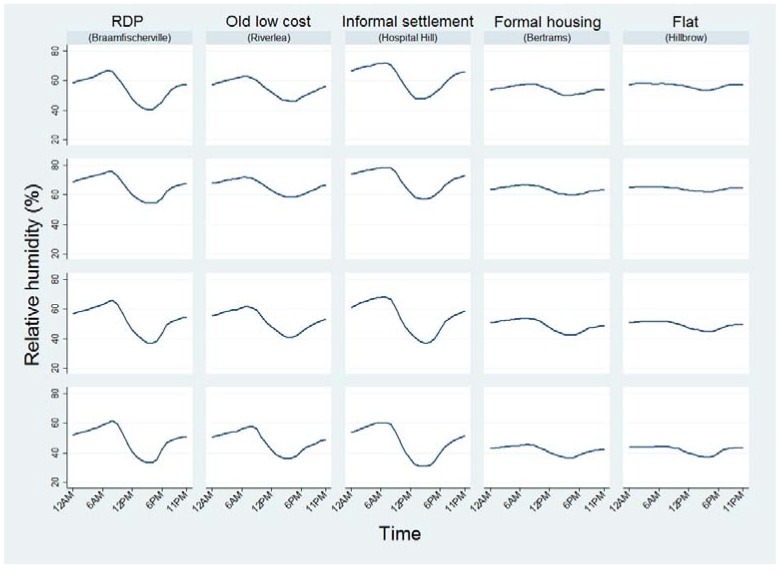
Mean relative humidity experienced throughout the day.

The greatest variation in humidity throughout the day occurred in informal settlements and RDP dwellings whilst the humidity remained relatively constant in flats and formal housing in Bertrams (Figure 6). In general, humidity was highest just after 06:00 and lowest during mid-afternoon. 

The results show carpets, brick walls and cement ceilings increased indoor and apparent temperature the most. Vinyl tiles, iron sheeting and the absence of a ceiling were found to decrease indoor and apparent temperatures the most (Table 5). 

It is interesting to note that the features contributing to higher temperatures are characteristic of flats (seen having the highest temperatures in Figure 2). Similarly, the features contributing to lower temperatures are characteristic of informal houses (seen having the lowest temperatures in Figure 2).

The monthly mean indoor temperatures and humidities by floor, wall and ceiling material are shown in Table 6a,b, Table 7a,b and Table 8a,b respectively.

There was also a significant difference in indoor temperatures between dwellings with and without ceilings for all months and areas (*p* < 0.001) (Table 8c).

## 4. Discussion

This study has shown the significant variation in indoor temperature across the main types of relatively low-cost housing in Johannesburg. Relative to the remaining suburbs, indoor temperatures in dwellings within Braamfischerville and the informal settlement of Hospital Hill varied most widely and were most sensitive to outdoor temperatures. 

Several factors influence indoor temperature in houses such as the size of the home, number of floors, doors and windows, shading, human behavior in relation to ventilation and heating/cooling etc. [32,33]. We looked at the role of housing type in the first instance as a factor influencing indoor temperature. Indoor temperatures were significantly higher than ambient temperatures across all housing types. However, the association between ambient and indoor temperature was strongest in RDP housing in Braamfischerville housing. Temperatures in apartments in Hillbrow correlated the least with ambient temperature.

As was the case for indoor temperature, indoor RH varied significantly across housing types, with RH being lower indoors than outdoors most of the time. Indoor RH levels ranged from approximately 42% in the month of May to more than 69% in March across the dwelling types studied. Indoor RH levels above 45–50% are known to be associated with increased prevalence of domestic dust mites [34] which are an allergen source causing allergic rhinitis and allergic asthma [35]. Dust mites may also invade and parasitize human tissues such as the gastrointestinal tract and lungs [36]. Elevated indoor RH is associated with microorganisms such as bacteria and mould [37]. For example, *Aspergillus versicolor*, a common indoor mould, will grow in RH levels of ≥65% [38] and is harmful to people who are immunocompromised and to those with existing lung illnesses [39,40].

The most notable difference in indoor temperature was seen in relation to the presence of ceilings. Indoor temperatures in Braamfischerville and Hospital Hill appeared to be most sensitive to ambient changes in temperature and RH. The lack of ceilings was the major difference observed between housing in Braamfischerville and Hospital Hill on one hand and housing in Riverlea and Hillbrow on the other. Braamfischerville residents have previously expressed dissatisfaction with their housing, with the lack of ceilings, dwelling size, overcrowding, and the lack of air bricks being amongst the main sources of discontent [12]. Indoor temperatures in RDP housing respond very quickly to outdoor temperature change when no ceilings are present [10], whereas the addition of a ceiling (retrofitted) greatly improves the thermal efficiency of dwellings [9]. 

Other factors affecting the thermal efficiency of post-1994 RDP housing are single wall thickness (15 cm) and the use of cement bricks, which allow high thermal conductivity between outdoor and indoor temperatures [10]. While all of the homes in the study had brick walls, the type of brick varied according to three types: cement bricks made from a mixture of cement and sand; clay bricks made from clay; and perforated hollow cement bricks [41].

There were several limitations in this study. The sample size was small and there was a 41% no response rate which impacted on the power of the study. Only one temperature data logger was placed in the living room in each dwelling. It should be noted that there is temperature variability between different rooms in a house and this should be accommodated for in future work. The study was conducted during relatively cool months, from the end of summer (February) to mid-autumn (May) and greater variation may have been seen during the very hot (December to January) and very cold months (June and July). Despite the measurements being made in late summer, very high temperatures in excess of 45 °C were measured in Hospital Hill in late February. Moreover, in the same suburb, temperatures below 7 °C were measured in May, indicative of temperature extremes being experienced in these homes.

In future studies more, detailed information such as the use of fans, lists of appliances contributing to the thermal environment in the home environment as well as assessing temperature-related health effects needs to be evaluated. 

Housing in South Africa is a constitutional right for all citizens however the quality of low cost housing creates further economic and social vulnerability because of poorly constructed dwellings. There needs to be a concerted effort to produce cheap but efficient and functional housing that will improve the quality of live for vulnerable impoverished communities.

## 5. Conclusions

Housing structure is one factor that affects indoor temperature and this has an impact on human health. In this study, RDP and informal dwellings were the most vulnerable structures in terms of indoor temperature stability and by inference, thermal comfort. These types of housing are common among the very poor and thus coping strategies in very hot and very cold environments will be limited and the risk of temperature-related diseases may be elevated.

## Figures and Tables

**Table 1:** Description of the study sites and housing types.

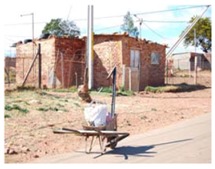	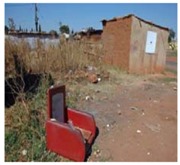	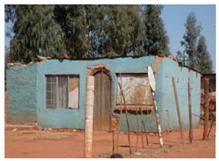	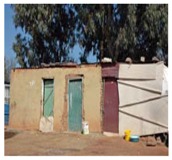
**HOSPITAL HILL** is an informal settlement near the western boundary of the City of Johannesburg. Informal housing in an informal settlement are typically made from a mix of materials including bricks, board, metal etc. Dwellings consisted of one to two rooms with no access to electricity and access to water is via a communal tap. The number of people residing in the dwelling range from 4 to 10 with a mean of 7.			
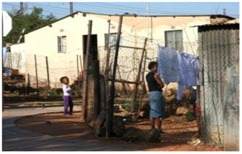	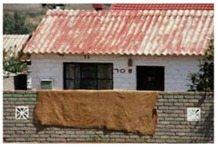	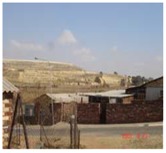	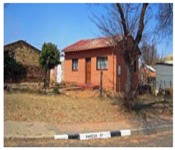
**RIVERLEA** is an apartheid era, low-cost housing development constructed in the early 1960s. Dwellings are constructed from bricks with asbestos ceilings and roofs. The number of people residing in the dwelling range from 1 to 11 with a mean of 8.			
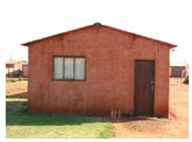	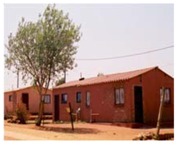	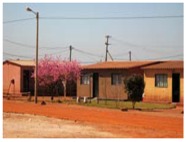	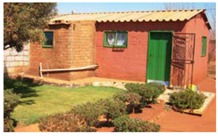
**BRAAMFISCHERVILLE** is a low-cost housing development built around 17 years ago following the transition from apartheid to democratic government in South Africa. Reconstruction and Development Programme (RDP] houses are typically made of bricks with a metal or asbestos roof. Generally, each dwelling consists of 2 rooms. The mean number of people in the dwelling was 7 with a range of 4–11.			
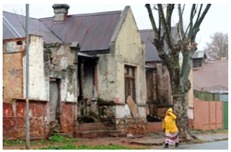	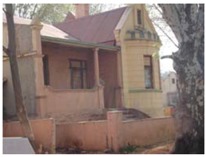		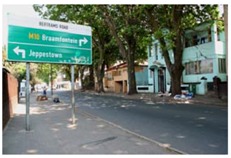
**BERTRAMS** is located to the east of central Johannesburg, and is one of its oldest suburbs in Johannesburg. Bertrams is currently a mixed commercial/residential inner-city suburb. The dwellings are a mix of low rise apartment buildings and standalone houses constructed from bricks with tiled or concrete roofs.			
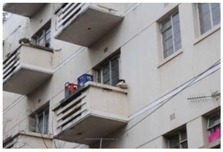			
**HILLBROW** is a high-rise, densely populated inner-city area. In the 1970s it was an Apartheid-designated ‘whites only’ area but by the 1980s had become a ‘grey area’, where people of different population groups and ethnicities lived. It acquired a cosmopolitan and politically progressive feel, but became characterised by degradation due to rapid population influx and growing neglect. The buildings are made from traditional/formal brick with flat concrete roofs and range from 5 to 15 floors. The mean number of residents per apartment is 1 with a range of 1–2.			

Photographs taken by author and colleagues.

**Table 2:** Building materials per housing type in the five different study sites according to respondents’ self-reported housing descriptions.

Housing Type	Exterior Walls: Main Construction Material per House	Floor: Main Type of Flooring Used	Ceilings Type	Roof Material	Mean Age of Dwelling in Years (Range)
Formal housing (Bertrams) *n* = 10	100% Bricks	10% cement 50% wood 40% tiles	100% ceiling boards	40% clay tiles 60% corrugated zinc roof (IBR) sheeting	55.5 (30–99)
Old pre-1994 low cost housing (Riverlea) *n* = 13	100% Bricks	31% cement 69% tiles	100% ceiling boards	15% corrugated zinc roof (IBR) sheeting 85% asbestos	49.8 (45–55)
Post-1994 low cost housing-RDP (Braamfischerville) *n* = 15	100% Bricks	67% cement 33% tiles	7% ceiling boards 93% no ceiling	100% asbestos	14.0 (11–18)
Apartments/Flats (Hillbrow) *n* = 13	100% Bricks	84% wood 8% tiles 8% vinyl	100% cement	100% Concrete	60.0 (60)
Informal settlement housing (Hospital Hill) *n* = 8	62% Bricks 25% Corrugated metal sheets 13% Dry wall	25% cement 12% tiles 38% vinyl 25% carpet	75% no ceiling 12% wood 13% boards	100% wood and corrugated zinc roof sheeting	7.3 (1–25)

**(a):** 

Month	February		March		April		May	
	Mean (SD)	Range	Mean (SD)	Range	Mean (SD)	Range	Mean (SD)	Range
Ambient	20.2 (0.7)	14.1–28.5	18.3 (1.4)	11.4–26.2	15.6 (1.7)	2.5–25.3	15.9 (2.3)	4.7–25.7
Braamfischerville (*n* = 15)	25.7 (4.2)	16.2–39.0	22.5 (3.7)	14.7–36.6	20.1 (4.5)	6.6–33.1	19.6 (4.7)	6.7–33.1
Riverlea (*n* = 13)	25.1 (2.2)	19.3–33.3	22.6 (2.2)	17.5–31.6	20.3 (2.8)	11.7–29.2	19.6 (3.1)	10.9–28.5
Hospital Hill (*n* = 8)	23.6 (4.3)	15.0–45.4	21.3 (3.9)	12.8–40.2	19.3 (4.8)	6.1–39.4	19.2 (5.2)	6.6–37.5
Bertrams (*n* = 10)	24.7 (2.1)	19.6–34.9	22.2 (2.1)	16.6–31.1	20.2 (2.2)	12.7–29.3	20.3 (2.2)	12.7–28.1
Hillbrow (*n* = 13)	24.4 (1.4)	19.3–28.7	22.5 (1.5)	17.4–30.3	21.5 (1.9)	15.9–28.5	22.3 (2.4)	16.9–30.7

**(b):** 

Month	February		March		April		May	
	Mean (SD)	Range	Mean (SD)	Range	Mean (SD)	Range	Mean (SD)	Range
Braamfischerville (*n* = 15)	27.5 (4.5)	16.7–43.8	24.4 (4.1)	15.1–39.0	20.1 (4.8)	4.6–34.6	19.2 (5.1)	4.8–34.2
Riverlea (*n* = 13)	26.8 (2.3)	20.1–35.7	24.5 (2.3)	18.0–33.5	20.4 (3.1)	10.1–29.9	19.2 (3.4)	9.3–32.1
Hospital Hill (*n* = 8)	25.4 (4.5)	15.1–49.7	23.0 (4.2)	12.8–42.4	19.2 (5.1)	4.7–40.9	18.6 (5.5)	4.2–37.0
Bertrams (*n* = 10)	26.2 (2.2)	19.4–36.6	23.7 (2.3)	17.3–32.7	20.0 (2.6)	11.1–30.6	19.6 (2.5)	10.5–28.9
Hillbrow (*n* = 13)	26.0 (1.6)	20.0–31.4	24.3 (1.6)	18.2–34.3	21.7 (2.2)	15.3–28.9	22.0 (2.7)	14.4–30.3

**Table 4:** Mean (standard deviation) and range in monthly indoor and ambient relative humidity (%) levels.

Month	February		March		April		May	
	Mean (SD)	Range	Mean (SD)	Range	Mean (SD)	Range	Mean (SD)	Range
Ambient	78.6 (8.1)	65.0–90.0	81.8 (10.3)	47.0–93.0	67.5 (16.9)	21.0–94.0	54.4 (17.2)	28.0–83.0
RDP houses (Braamfischerville, *n* = 15)	54.7 (11.3)	20.5–87.3	65.8 (13.1)	14.1–93.8	52.8 (13.2)	12.1–91.4	48.8 (12.9)	16.9–85.4
Low cost houses (Riverlea, *n* = 13)	54.9 (8.7)	27.9–79.7	65.5 (10.9)	21–93.9	52.1 (11.5)	14.3–92.4	47.6 (11.5)	14.3–93.5
Informal houses (Hospital Hill, *n* = 8)	61.5 (13.8)	18.3–87.9	69.4 (13.0)	16.2–93.4	54.7 (14.7)	10.1–88.4	47.4 (14.0)	13.1–84.2
Formal houses (Bertrams, *n* = 10)	54.1 (8.1)	21.7–73.4	63.4 (10.1)	19.9–84.5	49.0 (9.8)	15.2–75.8	41.7 (9.1)	13.3–66.4
Flats (Hillbrow, *n* = 13)	56.4 (6.6)	31.2–89.5	64.1 (8.9)	20.5–90.1	49.0 (10.1)	11.2–83.0	42.0 (10.0)	10.8–72.5

**Table 5:** Coefficients (95% CIs) from the multilevel linear regression of temperature and apparent temperature.

Housing Feature	Temperature (°C)	Apparent Temperature (°C)
**Floor Material**		
Tiles (reference)	0	0
Cement/Concrete slab	−0.2 (−0.8–0.5)	−0.3 (−1.0–0.5)
Wood	−0.5 (−1.4–0.3)	−0.9 (−1.8–0.1)
Vinyl tiles	−1.1 (−2.0–−0.1) *	−1.2 (−2.2–−0.1) *
Carpet	0.3 (−1.6–2.3)	0.3 (−1.9–2.5)
**Wall Material**		
Brick (reference)	0	0
Stone	−0.3 (−1.7–1.1)	−0.4 (−2.0–1.3)
Iron sheets	−1.8 (−3.5–−0.2) *	−2.4 (−4.2–−0.7) *
Dry wall	−0.9 (−2.7–0.9)	−0.9 (−2.9–1.1)
**Ceiling Material**		
Boards (reference)	0	0
No ceiling	−0.2 (−0.8–0.5)	−0.2 (−0.9–0.6)
Cement	1.0 (0.2–1.8) *	1.3 (0.4–2.2) *
**Model Constant**	21.2 (20.7–21.7) *	22.2 (21.6–22.7) *

* represents coefficients for which *p* < 0.001.

**(a):** 

Floor Materials	February	March	April	May
Cement/Concrete (*n* = 17)	24.9 (3.9) 16.0–39.0	22.2 (3.5) 13.8–36.6	20.0 (4.2) 6.6–33.1	19.7 (4.5) 6.7–33.1
Wood (*n* = 16)	24.5 (1.7) 19.3–34.9	22.4 (1.8) 17.1–31.1	21.1 (2.1) 12.7–29.3	21.5 (2.5) 12.7–30.7
Tiles (*n* = 19)	25.2 (2.7) 17.9–36.7	22.6 (2.5) 15.9–33.9	20.4 (3.1) 7.9–31.3	20.1 (3.5) 8.3–29.9
Vinyl tiles (*n* = 4)	24.2 (2.8) 18.4–35.4	21.8 (1.5) 18.4–26.9	19.7 (3.3) 9.3–30.8	19.6 (3.5) 9.5–30.4
Carpet (*n* = 1)	25.1 (1.6) 19.6–33.3	20.5 (5.2) 12.8–40.2	20.9 (1.4) 16.3–24.2	21.0 (1.3) 16.9–23.8

**(b):** 

Floor Materials	February	March	April	May
Cement/Concrete (*n* = 17)	56.1 (11.4) 20.5–86.6	66.1 (12.7) 14.1–93.9	51.7 (13.1) 10.1–92.4	46.4 (12.9) 13.1–93.5
Wood (*n* = 16)	55.5 (7.5) 21.7–89.5	63.5 (9.6) 19.9–90.1	48.3 (10.3) 11.2–83.0	41.6 (10.0) 10.8–72.5
Tiles (*n* = 19)	54.8 (9.2) 24.3–87.3	65.3 (10.8) 17.3–93.8	52.5 (11.3) 14.3–91.4	47.6 (11.5) 12.6–85.4
Vinyl tiles (*n* = 4)	59.4 (10.2) 28.5–84.6	68.5 (10.1) 23.7–91.0	55.9 (10.6) 15.7–82.3	43.1 (9.1) 21.4–61.1
Carpet (*n* = 1)	53.8 (7.4) 34.1–64.5	63.2 (9.8) 23.4–79.5	50.4 (9.8) 24.8–73.3	46.2 (17.6) 13.3–84.2

**(a):** 

Wall Materials	February	March	April	May
Stone (*n* = 2)	24.7 (2.0) 19.3–30.2	22.5 (2.0) 17.6–29.7	20.1 (2.6) 11.7–24.9	19.6 (3.0) 12.4–25.2
Brick (*n* = 53)	24.9 (3.1) 15.5–45.4	22.4 (2.8) 13.5–40.2	20.5 (3.4) 6.4–39.4	20.4 (3.8) 6.6–37.5
Iron sheets (*n* = 2)	22.1 (4.8) 15–36.1	20.0 (4.4) 12.8–33.6	18.0 (5.8) 6.1–33.2	17.9 (6.4) 7.3–33.7
Dry wall (*n* = 1)	24.4 (1.8) 21.2–29.4	21.8 (1.6) 17.8–26.4	19.6 (1.9) 14.4–24.2	19.4 (2.2) 14.4–25.1

**(b):** 

Wall Materials	February	March	April	May
Stone (*n* = 2)	55.3 (8.5) 31.2–70.5	65.3 (10.4) 24.4–91.6	51.8 (10.2) 18.7–78.8	45.0 (10.1) 15.7–67.5
Brick (*n* = 53)	55.6 (9.8) 18.3–89.5	65.2 (11.3) 14.1–93.9	51.3 (11.9) 11.2–92.4	45.6 (11.9) 10.8–93.5
Iron sheets (*n* = 2)	63.7 (16.8) 23.8–87.9	70.6 (16.2) 19.8–93.4	52.5 (18.7) 10.1–88.4	44.6 (18.0) 13.1–84.2
Dry wall (*n* = 1)	59.1 (6.9) 40.6–69.4	67.9 (7.2) 46.9–80.7	54.3 (7.7) 27.8–69.9	48.7 (8.5) 25.2–65.4

**(a):** 

Ceiling Materials	February	March	April	May
No ceiling (*n* = 20)	24.9 (4.5) 15.0–45.4	22.0 (4.0) 12.8–40.2	19.7 (4.8) 6.1–39.4	19.3 (5.1) 6.6–37.5
Cement (*n* = 13)	24.4 (1.4) 19.3–28.7	22.5 (1.5) 17.4–30.3	21.5 (1.9) 15.9–28.5	22.3 (2.4) 16.9–30.7
Wood (*n* = 1)	24.4 (1.8) 21.2–29.4	21.8 (1.6) 17.8–26.4	19.6 (1.9) 14.4–24.2	19.4 (2.2) 14.4–25.1
Boards (*n* = 23)	24.9 (2.3) 19.3–36.7	22.5 (2.3) 16.6–33.2	20.3 (2.7) 11.4–31.0	20.1 (2.9) 10.9–28.8

**(b):** 

Ceiling Materials	February	March	April	May
No ceiling (*n* = 20)	56.9 (13.0) 18.3–87.9	66.9 (13.5) 14.1–93.4	53.1 (14.1) 10.1–88.4	47.8 (13.6) 13.1–84.2
Cement (*n* = 13)	56.4 (6.6) 31.2–89.5	64.1 (8.9) 20.5–90.1	49.0 (10.1) 11.2–83.0	42.0 (10.0) 10.8–72.5
Wood (*n* = 1)	59.1 (6.9) 40.6–69.4	67.9 (7.2) 46.9–80.7	54.3 (7.7) 27.8–69.9	48.7 (8.5) 25.2–65.4
Boards (*n* = 23)	55.1 (8.9) 21.7–87.3	64.8 (10.8) 17.3–93.9	51.3 (11.2) 14.3–92.4	45.7 (11.3) 13.3–93.5

**(c):** 

Area	February		March		April		May	
Ceiling	Absent	Present	Absent	Present	Absent	Present	Absent	Present
RDP (Braamfischerville) *n* = 15	25.6 (4.2) 16.2–39.0	26.7 (3.7) 20.9–36.7	22.5 (3.8) 14.7–36.6	23.5 (3.4) 17.1–33.2	20.1 (4.5) 6.6–33.1	20.6 (3.9) 11.4–31.0	19.6 (4.8) 6.7–33.1	20.5 (3.6) 13.1–28.8
Informal settlement (Hospital Hill) *n* = 8	23.3 (4.8) 15.0–45.4	24.6 (2.1) 20.6–30.8	21.0 (4.2) 12.8–40.2	22.1 (2.1) 17.3–28.3	19.0 (5.3) 6.1–39.4	20.2 (2.4) 14.1–27.2	18.8 (5.8) 6.6–37.5	20.3 (2.8) 14.4–28.2
